# Desired Chinese medicine practitioner capabilities and professional development needs: a survey of registered practitioners in Victoria, Australia

**DOI:** 10.1186/1472-6963-8-27

**Published:** 2008-01-31

**Authors:** Charlie C Xue, Wenyu Zhou, Anthony L Zhang, Kenneth Greenwood, Cliff Da Costa, Alex Radloff, Vivian Lin, David F Story

**Affiliations:** 1 Division of Chinese Medicine, School of Health Sciences, World Health Organization Collaborating Centre for Traditional Medicine, RMIT University, Melbourne, Australia; 2 School of Health Sciences, RMIT University, Melbourne, Australia; 3 School of Mathematical and Geospatial Sciences, RMIT University, Melbourne, Australia; 4 Pro Vice Chancellor (Academic Services), Central Queensland University, Rockhampton, Queensland, Australia; 5 School of Public Health, La Trobe University, Melbourne, Australia

## Abstract

**Background:**

The State of Victoria in Australia introduced Chinese medicine practitioner registration in 2000 and issued its education guidelines in late 2002 for introduction in 2005. This study obtained practitioners' views on desired capabilities for competent Chinese medicine practice and to identify professional development needs.

**Methods:**

A questionnaire, consisting of 28 predefined capabilities in four categories with a rating scale of importance from one to five, was developed and sent to all registered Chinese medicine practitioners in the State of Victoria, Australia in October, 2005.

**Results:**

Two hundreds and twenty eight completed questionnaires were returned which represented a response rate of 32.5%. Of the four categories of capabilities, technical capabilities were considered to be the most important for clinical practice. Specifically, the ability to perform acupuncture treatment and/or dispense an herbal prescription was ranked the highest. In contrast, research and information management capabilities were considered the least important. The educational background of practitioners appeared to be an important factor influencing their rating of capabilities. Significantly, nearly double the number of practitioners with Australian qualifications than practitioners trained overseas valued communication as an important capability. For continuing professional education, clinical skills courses were considered as a priority while research degree studies were not.

**Conclusion:**

Registered Chinese medicine practitioners viewed skills training as important but did not support the need for research and information management training. This represents a significant hurdle to developing Chinese medicine as a form of evidence-based healthcare.

## Background

Chinese medicine (CM) is a unique medical system that has been taught and practised in China for more than 2000 years. Over the last several decades, CM has been increasingly used in the Western world, including Australia [1]. In parallel, CM education has been introduced in public and private institutions in a number of Western countries. In Australia, currently more than 20 institutions provide degree or diploma level education in CM [2]. Of these, four publicly funded Australian universities offer degree programs in acupuncture and/or Chinese herbal medicine. While the expansion of education programs in CM in the West has been welcomed, the consistency of educational quality has been an ongoing concern.

As a worldwide trend in medical education, there has been a growing emphasis on the learning process and learning outcomes, shifting from the traditional focus on course content and hours of tuition [3]. The required capabilities of practitioners have been used to guide curriculum design in most health practitioner education programs [4]. It is expected that by the time students graduate, they will have developed defined capabilities which meet the expectations of the public for safe and effective clinical practice.

Some medical schools in Australia have also introduced such capabilities into their curriculum. For example, the University of New South Wales has developed a new curriculum in which the capabilities of medical graduates are central to its design [5]. At RMIT University, all undergraduate and postgraduate programs are required to apply a capability-based curriculum approach. This approach was adopted in the design of the recently introduced postgraduate Chinese herbal medicine program in the School of Health Sciences [6].

Victoria is the only state in Australia, and also the first jurisdiction outside China, to have established and enforced standards of education and practice in CM. The Chinese Medicine Registration Board of Victoria (CMRBVic) has also published course approval guidelines for CM educational programs [7]. These guidelines use a traditional approach by describing the general outcomes of knowledge, skills and attributes of graduates of the approved courses, but they fail to define clearly demonstrable CM practitioner capabilities. Therefore, program approval is still largely based on general principles, rather than on more objective and measurable criteria. In addition, over nine hundreds of Chinese medicine practitioners were registered under much more flexible requirements between 1 January 2002 and 31 December 2004, known as the grand-parenting or transitional arrangement.

In developing the postgraduate Chinese herbal medicine program mentioned above, RMIT classified the Registration Board's guidelines [7] into four broad categories of desired capabilities: technical; communication; sustainability; and, research. A total of 28 capabilities were included under the four categories in a desired capability chart which was used to guide the development of the new masters program. Details of the process have been described elsewhere [6]. This capability chart was used as the basis for a bilingual (English/Chinese) questionnaire developed to seek practitioner views of the capabilities desired for Chinese medicine practice.

Variable qualifications and backgrounds are anecdotally presented among the current registered practitioners, while the CMRBVic education standards are based on what the profession has defined. This survey was to gather existing practitioners' views on desired capabilities of Chinese medicine practice against the CMRBVic requirements for new graduates to determine the knowledge and skills gaps between the two categories of practitioners. Consequently, professional development needs and strategies could be developed by the CMRBVic and/or the profession to narrowing down these gaps to ensure consistency of standards of practice.

## Methods

Human Research Ethics approval was obtained from the RMIT University's Human Research Ethics Committee prior to the commencement of the survey.

### Survey questionnaire

A CM practitioner capabilities chart was generated, based on the Chinese Medicine Registration Board's course approval guidelines in conjunction with a systematic review of Chinese medicine educational standards [7]. To ensure that the full breadth of CM capabilities was included, inputs from CM educators and regulators in Victoria were also sought through structured face-to-face interviews. A revised version of 28 capabilities was used in the development of the survey questionnaire. It comprised four categories of capabilities required for competent CM practice, namely, technical capability (TC), communication capability (CC), responsible and sustainable practice capability (RSC) and research and information management capability (RIMC). The questionnaire was translated into Chinese by academic staff at RMIT University who were native Chinese speakers.

### Survey participants

In October 2005, the bilingual questionnaire, with a plain language statement was sent to all registered CM practitioners in Victoria (*n *= 714). The plain language statement explained the purpose of the study, invited participation, and indicated that responses were confidential and that the data would be analysed and presented anonymously. A reminder letter was sent to all practitioners three weeks later to encourage participation. No further attempt was made to collect information from non-respondents.

Survey participants were asked to rate each of the 28 predefined capabilities in the questionnaire, using a five-point scale (i.e., 1 = not important, 2 = a little important, 3 = moderately important, 4 = important, 5 = very important). Additional capabilities or further comments could be added at the end of each of the four capability categories. Socio-demographic data on gender, age, educational background, and experience of practice were also collected.

### Data analysis

Data were entered into a Microsoft Excel spreadsheet and then analysed using the Statistical Package for Social Sciences (SPSS) for Windows, version 15.0. Chi-square analyses were used to compare participant responses across each capability. The significance level for multiple comparisons was adjusted by the Holmes-Bonferroni procedure [8].

## Results

### Demographic characteristics of survey participants

Two hundred and twenty-eight (228) completed questionnaires were returned, representing a response rate of 32.5%. Table 1 summarises the characteristics of survey participants. Nearly two-thirds (61.4%) were aged 35 – 54 years and just over half (55.8%) were males. Nearly one in five (19.2%) participants had received CM training in both Australia and overseas. In all, over two-thirds (70.1%) of participants had trained in Australia. Of these, almost three-quarter (71.3%) had been awarded at least a bachelor degree in CM. Among those who received CM training overseas (50.5%), approximately two-thirds (66.3%) had a bachelor degree or above. In addition, 41% of the participants had overseas practice experience. All except two participants (99.1%) had practiced in Australia. Of these, approximately two in five had practised for more than 10 years.

**Table 1:** Demographic characteristics of participating Chinese medicine practitioners (*n *= 228)

Characteristic		% (*n*)
Gender	Male	55.8 (125)
	Female	44.2 (99)

Age	18–34	24.7 (55)
	35–54	61.4 (137)
	55+	13.9 (31)

Australian Chinese medicine qualification	None	29.9 (67)
	< Bachelor	20.1 (45)
	≥ Bachelor	50.0 (112)

Overseas Chinese medicine qualification	None	49.5 (111)
	< Bachelor	17.0 (38)
	≥ Bachelor	33.5 (75)

Years of practice in Australia	Never	0.9 (2)
	< 10 years	56.1 (125)
	≥ 10 years	43.0 (96)

Years of practice overseas	Never	59.0 (131)
	< 10 years	25.7 (57)
	≥ 10 years	15.3 (34)

Data provided from the Chinese Medicine Registration Board of Victoria suggested that the basic demographic information of the survey participants did not differ appreciably from the overall profile of CM registrants. Specifically, males comprised 55.8% of the participants in the survey compared to 54.5% of CM practitioners registered by the Board (*p *> 0.05). In addition, the age profile of participants in the survey was almost identical to that of all CM registrants, there being, 24.7% and 23.6% aged 18 – 34, 61.4% and 60.3% aged 35 – 64, and 13.9% and 16.1% aged over 64, survey participants and all registered practitioners, respectively (*p *> 0.05).

### Participant responses to the 28 practitioner capabilities

Table 2 presents the mean score and standard deviation of participant responses to each of the 28 capabilities. Overall, 19 capabilities were rated 4.0 or higher (indicating the capability was considered to be important or very important). The mean scores for all technical capabilities (12 in total) were higher than four. Of these, the ability to perform acupuncture treatment and/or prescribe a Chinese herbal prescription were the highest (mean = 4.8). In contrast, all of the six capabilities under research and information management were rated lower than four, with the ability to develop a research protocol rated the lowest (mean = 3.1).

**Table 2:** Capabilities and survey participant scores

	**Capability**	**Mean (SD)**
**Technical Capabilities (TC)**		

TC1	Describe human structure and functions & their relevance to CM practice	4.33 (0.79)
TC2	Apply knowledge of Chinese and western medicine principles and diagnosis skills in diagnosis of disease	4.33 (0.89)
TC3	Formulate an appropriate CM prescription	4.75 (0.52)
TC4	Develop specific (individualised) treatment plans	4.73 0.48)
TC5	Diagnose and differentiate diseases/disorders according to both western and CM principles and techniques	4.34 (0.86)
TC6	Formulate a treatment plan including timelines for treatment and review	4.08 (0.85)
TC7	Give nutrition and dietary and preventive medicine advice in terms of CM knowledge for all areas of CM	4.01 (0.94)
TC8	Review and monitor patient's health and modify treatment accordingly	4.43 (0.71)
TC9	Refer to other practitioners, particularly medical practitioners, when appropriate in a timely manner	4.28 (0.86)
TC10	Perform acupuncture treatment and/or prepare and dispense a Chinese herbal prescription	4.76 (0.49)
TC11	Independently acquire technical knowledge about other diseases	4.25 (0.77)
TC12	Modify herbal formulae and/or treatment plan	4.13 (0.94)

**Communication Capabilities (CC)**		

CC1	Appropriately apply Chinese and western medical terminologies	3.93 (0.91)
CC2	Communicate effectively with patients and other health professionals	4.07 (0.86)
CC3	Refer patients to medical and other allied health professionals	4.00 (0.88)
CC4	Communicate effectively with fellow workers	3.99 (0.91)

**Response and Sustainable capabilities (RSC)**		

RSC1	Educate consumers of CM matters in order to promote sustainability	4.04 (0.93)
RSC2	Practise within regulatory/ethical/safety frameworks	4.62 (0.66)
RSC3	Remain financially viable	4.11 (0.96)
RSC4	Identify key business issues & draw on appropriate professional resources	3.75 (1.01)
RSC5	Participate to continue to learn (lifelong learning)	4.37 (0.79)
RSC6	Learn through experience (reflective learning)	4.62 (0.60)

**Research and Information Management capabilities (RIMC)**		

RIMC1	Keep up-to-date with CM research	3.94 (0.88)
RIMC2	Apply knowledge of methodological issues to CM clinical research	3.48 (1.00)
RIMC3	Critically review research publications relevant to CM	3.59 (0.93)
RIMC4	Apply knowledge in ethical issues surrounding CM research	3.67 (0.99)
RIMC5	Develop a research protocol	3.05 (1.09)
RIMC6	Disseminate research outcomes to different audiences	3.21 (1.02)

Note 1: all capabilities were rated on a five-point scale (i.e. 1 = not important; 2 = little importance; 3 = moderately important; 4 = important; 5 = very important)

Note 2: SD: standard deviation

### Items ranked the highest of importance

Table 3 summarises the 10 capabilities of the total of 28 with the highest scores on importance. These are broken down according to practitioners' demographic characteristics. Of these, seven were technical capabilities (TC) and three were responsible and sustainable practice capabilities (RSC). Among these highest-ranked capabilities, a higher proportion of female than male respondents considered TC5, TC8, TC10, RSC2 and RSC5 as "very important or important" (hereafter, important) (*p *< 0.05). On the other hand, a significantly lower proportion (75.4%) of participants aged 35 – 54 considered the skill to diagnose and differentiate diseases according to both western and CM principles and techniques (TC5) to be important, compared with younger participants (aged 18 – 34, 92%, *p *< 0.05) and older participants (55+, 90%, *p *< 0.05).

**Table 3:** Capabilities ranked of high importance

**Demographic information**		**Percentage of practitioners rating capability as important or very important**									
		
		**TC10**	**TC3**	**TC4**	**RSC6**	**RSC2**	**TC8**	**RSC5**	**TC5**	**TC2**	**TC1**
Gender	Male	95.8	96.6	97.5	93.6	88.8	82.2	80.8	75.9	81.7	80.0
	Female	100	97.0	100	93.9	98.0*	96.8*	90.8	89.2	86.7	86.9

Age	18–34	95.9	96.4	100	98.2	92.7	94.0	92.7	92.0	87.3	87.3
	35–54	99.2	97.0	98.4	92.7	92.6	86.8	83.8	75.4	81.3	80.6
	55+	90.3	96.6	96.8	93.5	93.5	83.3	80.6	90.0	82.1	89.7

Australian CM qualification	None	97.0	97.0	97.0	85.1*	88.1	70.8*	70.1*	81.8	81.8	77.3
	< Bachelor	100	95.2	100	95.6	97.8	97.7	93.3	76.2	83.3	90.5
	≥ Bachelor	97.1	97.3	99.0	98.2	93.7	96.2	91.0	84.2	85.5	83.8

Overseas CM qualification	None	98.0	97.2	99.0	97.3	95.5	97.1*	91.8	79.2	85.2	86.1
	< Bachelor	97.3	94.7	97.3	89.5	92.1	83.8	81.6	88.9	78.4	73.7
	≥ Bachelor	97.3	97.3	98.6	90.7	89.3	79.2	77.3*	81.9	84.9	83.6

Years of practice in Australia	< 10 years^†^	96.6	98.4	99.2	96.1	95.2	93.2	90.5	84.3	85.4	88.7
	≥ 10 years	97.8	94.7	97.8	91.7	89.6	81.3	79.2*	78.0	79.8	76.6

Years of practice overseas	Never	97.5	96.9	99.2	98.5*	96.2	96.6*	93.8*	82.8	83.5	86.7
	< 10 years	98.2	96.4	100	89.5	86.0	78.9	71.9	75.0	78.6	75.0
	≥ 10 years	93.9	97.0	93.9	85.3	91.2*	71.9	76.5	87.9	87.9	87.9

*: Significant difference between groups (via χ^2 ^test, significance level for multiple comparisons was adjusted by Holmes-Bonferroni procedure).

†: Including participants never practiced in Australia (*n *= 2)

Participants with at least one Australian CM qualification were more likely to value lifelong learning (RSC5). The majority of people with an Australian CM qualification (91.0% of those with a bachelor degree or above and 93.3% of those with less than a bachelor degree) considered lifelong learning to be important. This proportion was significantly lower among those without any Australian CM qualifications (70.1%, *p *< 0.001, Table 3). Supporting this finding, over nine-tenths (91.8%) of respondents without an overseas qualification but with an Australian qualification, considered lifelong learning to be important, in comparison to those respondents holding an overseas qualification (81.6% among people with less than a bachelor degree or 77.3% among those with a bachelor degree or higher, *p *< 0.05). Similarly, significantly lower proportions of respondents without an Australian qualification appreciated the importance of learning through experience (RSC6) and the ability to review and monitor a patient's health and modify treatment accordingly (TC8, Table 3).

CM practice experience in Australia and/or overseas appears to have influenced practitioner attitudes regarding the importance of each capability. Compared to those with overseas CM experience, participants who had never practised overseas gave significantly higher ratings to four capabilities (TC8, RSC2, RSC5 and RSC6). For example, TC8, the capability to review and monitor a patient's health and modify treatment accordingly, was considered important by 96.6% of practitioners without overseas experience, compared with only 78.9% of practitioners who had less than 10 years overseas experience and 71.9% of practitioners who had 10 years or more overseas experience (*p *<0.001, Table 3).

### Items ranked lowest on importance

The ten capabilities that were rated lowest on importance comprised all six items in the research and information management category (RIMC), three out of the four communication capabilities (CC1, CC3 and CC4) and one of the responsible and sustainable capabilities (RSC4, Table 4). None of the technical capabilities was in the list of "lowest-ranked" capabilities.

**Table 4:** Capabilities ranked of low importance

**Demographic information**		**Percentage of practitioners rating capability as not important, less important or moderately important (%)**									
		
		**CC3**	**CC4**	**RIM C1**	**CC1**	**RSC4**	**RIMC4**	**RIMC3**	**RIMC2**	**RIMC6**	**RIMC5**
Gender	Male	34.7	32.3	28.2	35.2	38.7	48.4	46.8	49.2	53.7	66.9
	Female	19.2*	25.3	30.6	25.3	37.4	43.9	49.5	50.5	66.3	70.1

Age	18–34	16.7	18.2	30.2	18.2	18.5*	39.6	39.6	39.6	47.2	67.9
	35–54	29.2	31.6	26.7	35.8	44.5	47.8	51.1	52.3	64.2	71.6
	55+	38.7	38.7	36.7	29.0	48.4	46.7	46.7	53.3	60.0	53.3

Australian CM qualification	None	53.7*	59.7*	39.4	44.8	58.2*	58.5	62.1	53.8	64.6	66.7
	< Bachelor	15.6	13.6	24.4	28.9	31.1	40.0	51.1	52.3	62.2	73.3
	≥ Bachelor	17.1	17.0	25.2	23.2*	28.8	42.0	38.4*	46.4	55.0	67.3

Overseas CM qualification	None	14.5*	18.0*	26.6	27.0	30.0	39.1	40.0	48.6	57.4	72.2
	< Bachelor	39.5	42.1	28.9	39.5	50.0	54.1	50.0	56.8	60.5	65.8
	≥ Bachelor	41.3	39.2	33.3	32.0	44.0*	53.3	58.7	48.0	61.3	64.0

Years of practice in Australia	< 10 years^†^	27.0	26.8	26.6	26.0	30.0*	39.8	41.9	44.3	58.9	67.5
	≥ 10 years	28.1	32.6	31.9	36.5	49.0	53.2	55.3	55.9	60.2	69.1

Years of practice overseas	Never	15.4*	15.4*	24.2	24.4	30.0*	39.8	41.4	47.6	56.2	68.5
	< 10 years	47.4	43.9	36.8	42.1	47.4	56.1	54.4	52.6	64.9	75.4
	≥ 10 years	41.2	58.8	34.4	35.3*	58.8	51.6	62.5	51.6	64.5	56.3

*: Significant difference between groups (via χ^2 ^test, significance level for multiple comparisons was adjusted by Holmes-Bonferroni procedure).

†: Including participants never practiced in Australia (*n *= 2)

In relation to socio-demographic data, the only gender difference in responses to the ten "lowest-ranked" capabilities was for CC3 (refer patients to medical and other allied health professionals) where over one-third (34.7%) of male respondents considered this capability as not important/less important or moderately important (hereafter, "less important"), whereas only one in five (19.2%) of female respondents considered it to be less important (*p *< 0.05).

In relation to age, younger participants (aged 18 – 34), those with Australian qualifications, without overseas qualifications and with less than 10 years clinical experience in either Australia or overseas, were less likely to consider RSC4, an ability to identify key business issues and draw on appropriate professional resources, to be less important. Specifically, a lower proportion (18.5%) of those aged 18 – 34 considered RSC4 to be less important, compared with those aged 35 – 54 (48.4%) and those aged 55 or older (44.5%, *p *< 0.005, Table 4).

In relation to practice experience, significant variations were observed in the perceived importance of communication capabilities. The capabilities to appropriately apply Chinese and Western medical terminologies (CC1), to refer patients to other health professionals (CC3) and, to communicate effectively with fellow workers (CC4), were more likely to be considered as less important by participants who had overseas experience (ranging from 35.3% – 58.8%), while a significantly lower proportion of respondents who had no overseas experience fell in the same category (ranging from 15.4% – 24.4%, *p *< 0.005, Table 4). In addition, over half (53.7% – 59.7%) of respondents without Australian CM qualifications considered these capabilities (CC3 and CC4) to be less important, while these proportions decreased to some 14% – 17% among respondents with at least an Australian CM qualification (*p *< 0.001, Table 4).

As noted above, research and information management capabilities overall, were considered less important compared to the other three domains of capabilities, across participants with different demographic characteristics. The only exception was found for RIMC3, a capability to review critically research publications relevant to CM. Specifically, nearly two-thirds (62.1%) of respondents without an Australian CM qualification considered RIMC3 to be less important, whereas approximately half (51.1%) of respondents with less than an Australian bachelor degree and just over one-third (38.4%) of respondents with an Australian bachelor degree or higher, responded in the same way (*p *< 0.05).

### Professional development needs

Practitioners were asked about their plans and need for professional development in the next five years. Nearly two-thirds (61.9%) of participants indicated a need for a short course in CM to update their clinical knowledge and skills. About half (44.6%) indicated that they would like to undertake research studies to specialise in one or more areas to enhance practice. More than one-third (37.4%) considered undertaking postgraduate studies to gain future qualifications. Undertaking short courses in Western medical sciences were also considered by nearly one-quarter of the participants (23.4%).

The number of years of overseas practice experience and having an Australian qualification were significant factors that contributed to participants' intention to pursue professional development. Those who had never practised overseas were more likely to want to pursue postgraduate studies to gain further qualifications (45.8%) than those with less than 10 years overseas experience (31.6%, *p *< 0.05) and those that had more than 10 years overseas experience (15.2%, *p *< 0.05). However, compared with the latter groups, participants without overseas experience were less likely to want to undertake postgraduate research studies (30.5%, 66.7% and 63.6%, respectively, *p *< 0.05). Similarly, those who had at least one Australian CM qualification were more likely to undertake postgraduate studies (43.5%) than those without Australian qualification (23.4%, *p *< 0.05), but less likely to pursue research studies (36.4% vs. 65.6%, p < 0.05). Further, over half (56.4%) of participants aged 18 – 34 would like to engage in postgraduate studies, while fewer than one-third (32.8%) of those aged 35 – 54 and less than one-quarter (23.3%) of those aged 55 or older, responded similarly (*p *< 0.01).

## Discussion

There is no existing literature on practitioners' view of the importance of desired CM professional capabilities. The current study surveyed all registered CM practitioners in the State of Victoria, Australia to obtain existing practitioner views regarding desired capabilities and to identify their continuing educational needs. The findings of this study may inform the development of CM educational programs, and are of relevance to regulators who are increasingly concerned about continuing mandating professional development (such as the Victorian Health Professions Registration Act 2005) [9] and ensuring that health professional education reflects contemporary requirements of a changing health industry [10]. These findings may also be relevant to a broad range of health professionals as well, beyond CM practitioners.

The response rate of this study was 32.5%. The mailing list used in the current survey was provided by the Chinese Medicine Registration Board of Victoria and hence. Hence, the sampling frame was the totality of eligible population. Perhaps, the most noteworthy shortcoming of the current survey is that two-thirds of the targeted population did not respond. This may have introduced a non-response bias towards the rating of capability items. Despite the fact that the study sample was comparable to the target population on important characteristics (see Methods), interpretation of the findings from this study needs caution and a follow-up study is required with an effort to increase the response rate. The other issue is to clarify the meaning of postgraduate studies as it appeared to be understood differently by participants

It is understood that effective clinical practice is dependent on graduates' ability to integrate and consistently apply a number of capabilities beyond profession-specific skills and knowledge [11]. Thus, it is not surprising that technical capabilities (specifically, the ability to perform acupuncture treatment and/or dispense an herbal prescription) were considered to be the most important aspects of clinical practice, followed by responsible and sustainable practice capabilities. As a clinically oriented workforce, practitioners seek to learn their trade. In contrast, most participants considered the ability to develop a research protocol, an element of the research and information management capabilities, to be only moderately important. Such views reflect the theoretic underpinning of Chinese medicine held by members of the profession, something that needs to be addressed through continuing professional education and incorporation of the importance of this activity into CM practitioner education. Nevertheless, in recent years, there has been an increasing body of scientific evidence of CM treatment from rigorously designed clinical research which may serve as the base of integrative health care.

The country where practitioners' obtained their CM education appears to be an important factor influencing their rating of the importance of a number of capabilities, in particular, communication capabilities. A much lower proportion of those without Australian qualifications considered communication capabilities to be important. This may be related to socialisation during the course of education, which in turn reflect differences in the value orientation in health professional education generally or in specific curriculum design [12]. At the same time, it might also reflect self-selection, that is, Australian trained CM practitioners choose to enter the profession because they consider it to offer a holistic approach to health-care in contrast to conventional health-care professions. These findings are consistent with a previous study comparing CM tertiary education in Australia and China. They suggest that, CM education in Australia shares a number of common features with that in China, but the location of education has an impact on its curriculum design as well [12]. This is a matter that warrants additional exploration.

In relation to the "less important" capabilities, over one-third of male participants considered patient referral capability to be less important. This is a finding that will be of concern to health policy-makers, in the context of ensuring continuity of care and the most appropriate care. New graduates rated higher identifying key business issues than did elderly practitioners. This may reflect their educational background and the early stage of their practice development. However, it is clear that participants without overseas experience would like to gain further qualifications, but were less willing to engage in research studies. In addition, participants with Australian qualifications would like to pursue postgraduate study, but not research. The motivation underlying selection of a particular course for professional development warrants further study.

Much educational research has been done for the western medicine profession, however, in the USA, the concept of embedding desired essential capabilities into curriculum design is a recent development [13]. These essential capabilities are broadly similar to the capabilities included in the current study. For example, clinical (technical) skills, communication skills, information management and critical thinking and research have all been addressed. It has been stated that such core capabilities [14] would help to determine what teachers are supposed to teach, what students are expected to learn, and what educational experiences all physicians must have.

It is challenging to identify a range of capabilities that truly cover the roles of CM practitioners in the Australian health-care setting. Unlike the situation in Asian countries and a limited number of Western countries, CM practitioners in Australia are mainly self-employed. Compared with the traditional approach to curriculum design, the capability-based approach of professional education potentially leads to individualised flexible learning, transparent standards, and increased public accountability [15]. Other studies have demonstrated that medical residents who had attended courses based on competencies perform better and are safer practitioners [16]. In Australia, criterion-referenced approaches to set standards have been used to define and measure competencies for graduate entry medical programs [17]. A sophisticated model of professional education is required that recognises both basic standards and continuing professional development [18] and thus, to enable Australian CM practitioners to provide a safe and effective health service.

## Conclusion

This study provides an in-depth perspective of the professional capabilities of CM practitioners – a profession with increasing numbers of practitioners in Western countries, including Australia. The views on capabilities reported by current practitioners can inform educational curriculum design. However, the lack of recognition of the importance of research capability will continue to hinder the development of CM as an evidence-based healthcare profession.

## Competing interests

The author(s) declare that they have no competing interests.

## Authors' contributions

The following authors contributed to the conceptualisation of the project: CX, WZ, AZ and AR. The following authors contributed to the data collection, analysis and interpretation: CX, WZ, AZ and CD. The following authors contributed to the draft of the manuscript: CX, WZ and AZ. The following authors contributed to the review and commentary on the manuscript: KG, VL, AR and DS. All authors read and approved the final manuscript.

## Pre-publication history

The pre-publication history for this paper can be accessed here:


